# Spatial processing and visual backward masking

**DOI:** 10.2478/v10053-008-0016-1

**Published:** 2008-07-15

**Authors:** Michael H. Herzog

**Affiliations:** Laboratory of Psychophysics, Brain Mind Institute, Ecole Polytechnique Fédérale de Lausanne (EPFL), Switzerland

**Keywords:** temporal processing, verniers, unmasking, metacontrast

## Abstract

Most theories of visual masking focus prima-rily on the temporal aspects of visual information processing, strongly neglecting spatial factors. In recent years, however, we have shown that this position is not tenable. Spatial aspects cannot be neglected in metacontrast, pattern and un-masking. Here, we review these results.

## INTRODUCTION

In visual backward masking, perception of a target is impeded by a trailing mask. Most research has focused on the phenomenon of B-type masking, in which the strongest deterioration of performance occurs for intermediate SOAs. In these investigations, usually metacontrast masks are used, i.e. masks not overlapping with the target. Deteriorated performance is often explained by neural inhibitory mechanisms such as lateral inhibition (e.g. Bridgeman, 1971; Growney & Weisstein, 1972), mask blocking (Francis, 2000), dual channel inhibition (e.g. Breitmeyer & Ganz, 1976; Öğmen, 1993), delayed facilitation (e.g. Bachman, 1994), contour elimination (e.g. Kolers, 1962; Werner, 1935), or object substitution (e.g. Di Lollo, Enns, & Rensink, 2000). For example, in the influential models by Breitmeyer and Ganz (1976) and Bachman (1994), target and mask processing occurs in two channels, a faster and a slower one, thereby allowing the mask signal in the faster channel to catch up with the target signal in the slower channel (Öğmen, 1993). 

In A-type masking, performance improves mono-tonically when the ISI between the target and mask increases. The effects of the mask on the target are often explained in terms of contrast reduction (e.g. Eriksen, 1966) or camouflage (e.g. Coltheart & Arthur, 1972; Enns, 2004).

Almost all studies of both A and B-type masking have a common focus on the temporal characteristics of the target and mask, largely neglecting non-basic spatial dimensions (however, see Werner, 1935; Williams & Weisstein, 1981, 1984). Here, we review results suggesting that the spatial layout of the target and mask exerts a tremendous influence on backward masking that was largely neglected previously. In particular, spatial grouping seems to be a key factor for certain masking effects. We will argue that, for this reason, models have to incorporate explicit spatial processing components. Models employing temporal mechanisms only are not sufficient.

## RESULTS

### Pattern and A-type masking

In pattern masking (by structure), mask and target spatially overlap. Usually A-type masking is found, which is explained, in terms of integration masking, for example, as a result of luminance summation and contrast reduction (e.g. Eriksen, 1966), by camouflage and montage (recently, Enns, 2004), or a degraded target image (e.g. Scheerer, 1973). These factors are often assumed to occur at stages as early as the retina (e.g. Michaels & Turvey, 1979).

**Figure 1.**
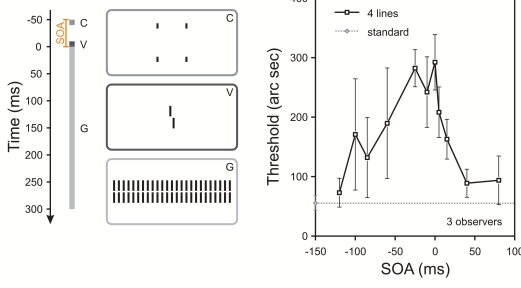
A left or a right offset vernier (V) was presented for 10ms and followed immediately by a grating comprising 25 aligned verniers (G) lasting for 300 ms. Observers had to indicate the offset direction of the vernier in a binary task. The horizontal line in the results graph indicates the threshold in this condition (“standard”). In addition to the standard grating, four contextual lines could be displayed with varying SOAs in relation to the vernier onset (SOA denotes the onset asynchrony of contextual lines (C) relative to the standard grating). These lines appeared above or below the third grating element to the left and right of the center. Lines were separated by a small vertical gap of 200’’ from the grating and presented for 5 ms or 10 ms (a SOA of -50ms is shown in the stimulus sketch). Performance strongly deteriorated for SOAs from -100 ms to 30 ms, i.e. much longer than the duration of the four lines. Reprinted from Vision Research, 43, Herzog M.H., Schmonsees U., & Fahle M., Timing of contextual modulation in the shine-through effect, 2039-2051 (2003a), with permission from Elsevier, where further experimental details can be found.

In a series of experiments using pattern masks, we have shown that these explanations are not sufficient (Herzog & Fahle, 2002; Herzog, Fahle, & Koch, 2001; Herzog & Koch, 2001). Figure 1 shows a typical example of these experiments. A vernier target is followed by a grating comprised of 25 aligned verniers; a moderate threshold elevation occurs compared to when the vernier is presented without the grating. This masking can be strongly potentiated if four single contextual lines are presented in addition to the grating: the vernier target can be rendered invisible and thresholds dramatically rise (Figure 1).

**Figure 2.**
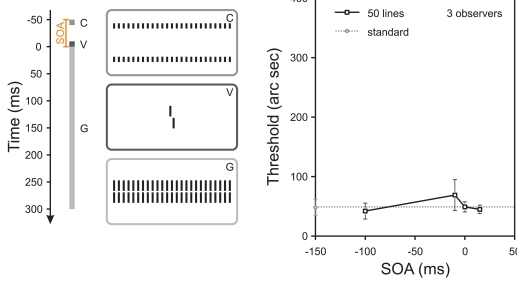
Same experimental condition as in figure 1 except that instead of single contextual lines, contextual gratings were presented including the single contextual lines from the previous figure. Performance is only slightly elevated independent of SOA. With permission from Herzog, Schmonsees, & Fahle (2003a) (see figure 1).

This interference is dominant in a temporal window of more than 100 ms and can hardly be explained with the classical accounts of integration masking. Luminance summation and contrast reduction may play a role if only the central grating follows the vernier (horizontal line in Figure 1). However, they cannot explain why adding four additional lines potentiates masking. This becomes even more evident when taking into account that adding 2*25 contextual lines, hence further increasing energy, undoes the masking of the four lines which are contained in the 2*25 lines (Figure 2). Camouflage or montage play no role since the four lines may even serve as a reference to localize the vernier (collinear lines above and beneath the central grating element also yield a strong performance deterioration; Herzog, Schmonsees, & Fahle, 2003b). Finally, the vernier is covered only by the central grating element in all conditions, which yields the same degree of image distortion in the near neighborhood of the vernier. Still, performance varies strongly with the spatial layout of the contextual elements. Taken together, classical explanations of integration masking fail to account for our masking results (Herzog, Dependahl, Schmonsees, & Fahle, 2004; Herzog & Fahle, 2002).

**Figure 3.**
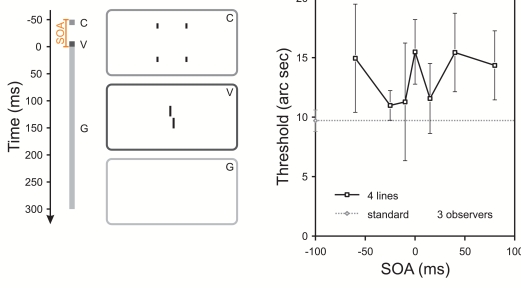
Same experimental condition as in figure 1 except that no standard grating was presented, i.e. only the four contextual lines and the vernier target. Performance is only slightly elevated. Please note the change of scale of the ordinate. With permission from Herzog, Schmonsees, & Fahle (2003a) (see figure 1).

It is interesting that masking is not linear regarding the masking of the mask pieces. The four contextual lines themselves exert only weak masking (by a factor of about 1.5; Figure 3) while the grating presented without these lines causes a threshold elevation of a factor of 5.5 (Figure 1, horizontal line). However, if the grating and contextual lines are displayed together, the vernier is largely invisible and thresholds can be elevated by more than a factor of 31 (see Figure 2). These non-linear results show that the strength of a pattern mask cannot simply be explained by the masking of its parts.

Our results clearly show that explanations of pattern masking have to carefully consider aspects of the spatial layout of the target and mask. We could show that some of the above results can be reproduced with a simple but dynamic model of spatial information processing (Hermens & Ernst, this volume; Herzog, Ernst, Etzold, & Eurich, 2003).

It is very important to note that the dramatic changes of performance, caused by rather simple spatial manipulations, occur only in a short temporal window. Even only slightly longer vernier durations, as used above, yield weak or no masking independent of the spatial layout (e.g. Herzog, Schmonsees, & Fahle, 2003a). Hence, it seems that the above results reveal complex spatio-temporal effects at the very beginning of spatial information processing.

### Unmasking

The previous subsection suggests an important role for the spatial layout of the mask in pattern masking. In this subsection, we show analogous results for unmasking. In unmasking, a target is followed by two masks. Under some conditions, performance is better in the two-mask condition compared to when only the first mask is presented. Hence, the second mask unmasks the first one in some way (e.g. Amassian, Cracco, Maccabee, Cracco, Rudell, & Eberle 1993; Breitmeyer, Rudd, & Dunn, 1981; Briscoe, Dember, & Warm, 1983; Öğmen, this volume; Robinson, 1966; Tenkink, 1983).

**Figure 4.**
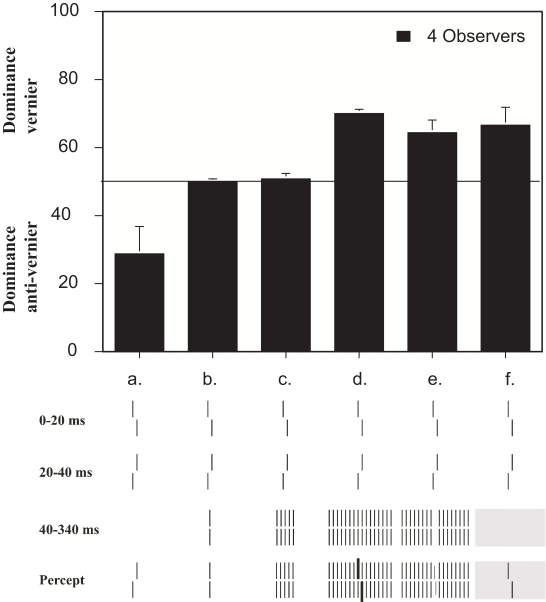
A vernier was followed by an anti-vernier (a) which in turn could be followed by an aligned vernier (b), a grating with 5 or 25 aligned verniers (c, d), a metacontrast grating, or a light field (f). Gratings lasted for 300 ms, verniers for 15 ms or 20 ms. The metacontrast grating resulted from removing the central element in the 25-element grating. If only the vernier and anti-vernier were presented, the anti-vernier dominated performance, indicated by a value below 50%. For a single aligned vernier or a 5-element grating no obvious dominance occurred, whereas extended masks led to unmasking: the vernier dominated (performance was above 50%). From Herzog, Lesemann, & Eurich (2006) with permission from “Advances in Cognitive Psychology”.

Using a feature fusion paradigm, we have shown how the spatial layout contributes to unmasking. We presented a vernier followed by a second vernier with the same duration and spatial parameters as the first vernier except for having an offset with opposite direction (Herzog, Parish, Koch, & Fahle, 2003). This “anti-vernier” serves as the first mask. With this condition, both verniers fuse yielding the percept of one single vernier. The anti-vernier dominates performance more strongly than the vernier, i.e. backward masking is stronger than forward masking (Figure 4a). When these two verniers are followed by an additional mask, dominance can reverse, i.e. the vernier dominates performance (Figure 4d-f; Herzog, Lesemann, & Eurich, 2006). However, this unmasking is present only for extended masks but not, for example, for a single aligned vernier, even though this single vernier is part of the 25-element grating which yields strong unmasking (Figure 4b,d. On the other hand, the single vernier is not part of the metacontrast grating which, however, yields unmasking like the 25-element grating. Hence, unmasking like pattern masking cannot be explained by the masking of its parts. This again suggests complex spatial processing.

### Metacontrast and B-type masking

B-type masking is usually believed to be the most interesting phenomenon in backward masking. A later presented mask can catch up to an earlier presented target and dominate performance, thereby ruling out an ultra-fast feedforward processing as found in other domains of vision (e.g. Thorpe, Fize, & Marlot, 1996; VanRullen, this volume). It should be mentioned that B-type masking loses much of its mystery when it is accepted that the brain acts as a temporal low pass filter and some temporal non-linearities are involved (Francis, 2000; Francis, this volume; Francis & Herzog, 2004). Here, we show that temporal aspects are not the whole story but that B-type masking strongly depends on the spatial layout of the mask and target.

**Figure 5.**
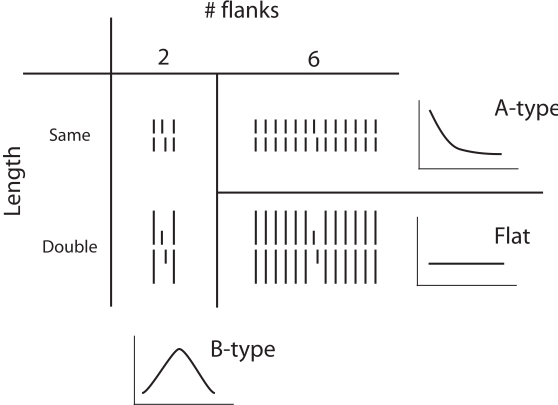
A vernier was flanked by either 1 or 6 lines on each side having either the same length or a length twice as long as the vernier. With the single flanks (# flanks 2), B-type masking occurs for both lengths. With 6 flanking lines on each side, strong A-type masking occurs for equal length flanks and an almost flat masking function for the double length flanks with thresholds only slightly above the one for an unmasked vernier. This figure summarizes results which cover four figures in Duangudom, Francis, & Herzog (2007). Stimulus examples sketch the condition for an SOA of 0 ms, i.e. simultaneous presentation. The vernier target is always the center element.

A vernier was presented for 20 ms and flanked by a line on each side, presented for 20 ms as well. Flank length was either the same as the vernier or twice as long. These metacontrast masks exerted B-type masking as expected (Figure 5; Duangudom, Francis, & Herzog, 2007; see also Otto, Öğmen, & Herzog, 2006; Otto, this volume). Surprisingly, for more flanking lines, A-type masking or flat masking functions were obtained depending on the length of flanks. Hence, we can change the masking function, e.g. from A to B, by changing the spatial layout of the mask. Surprisingly, the weakest masking was obtained for the mask with 6 lines on each side of the vernier being twice its length (Figure 5; Duangudom, Francis, & Herzog, 2007). This mask has the highest energy but still yields the weakest masking contrary to many models of masking (Breitmeyer & Öğmen, 2006, p. 48).

## DISCUSSION

Visual masking has been explored for more than a century. Still, the underlying mechanisms are subject for debate. Most models try to explain masking from purely temporal grounds, ignoring spatial aspects (only the model by Francis, 1997, has a full 2-dimensional spatial representation). Here, we have provided strong support for the involvement of spatial aspects in pattern, un-, and metacontrast masking. These effects are only visible with backward masking in a very narrow time window. For example if an ISI of 10 ms only is inserted between the vernier and the standard grating, adding contextual lines raises thresholds only modestly. Hence, contextual modulation has vanished (Herzog et al., 2003a; see also Herzog, Koch et al., 2001). We believe that masking with the shine-through effect reveals aspects present only at the very beginning of spatial information processing.

**Local contour interactions**. B-type masking with metacontrast masks is often assumed to occur because the mask inner contour suppresses the processing of the target contours (e.g. Werner, 1935). In support of this hypothesis, it was found that the larger the space between the target and the inner contour of a metacontrast mask, the better was the performance (e.g. Growney, Weisstein, & Cox, 1977; Kolers, 1962; review: Breitmeyer & Öğmen, 2006, p. 56). Hence, local spatial interactions seem to be important.

However, contrary to this proposition, we could change the masking function qualitatively from B-type to A-type masking while leaving the inner contour of the metacontrast masks constant (Figure 5). Thus, local computations between the target and the neighboring masking elements are not sufficient to explain B-type masking.

In pattern masking, we used gratings. Performance changed greatly in the various conditions even though the standard grating was constant (see Figure 1). In unmasking, both the 25-element and the metacontrast grating yielded comparable results whereas the proximity of contours clearly differed in these conditions. For these reasons we deny an important role of local contour interactions, at least with our stimuli. Also, models based on *simple* lateral interactions may not be able to explain many of our results.

**Energy ratio**. Another aspect considered important to masking is the energy ratio between the target and mask. For example, it was often proposed that B-type masking occurs only when the target and mask have approximately the same energy or when the mask has weaker energy than the target (e.g. Breitmeyer & Öğmen, 2006, p.48). Energy is usually defined in terms of the product of luminance and duration of the elements of the mask. The mask blocking idea by Francis (2000) has provided an elegant mathematical description for this argument that can be sketched as follows: For SOA = 0 ms, a strong target “blocks” the mask completely. For intermediate SOAs, the target signal has decayed and the mask can influence performance. For longer SOAs, the mask arrives too late to influence target processing. Hence, masking is strongest for intermediate SOAs.

However, the target-to-mask energy does not play a role in our experiments either in pattern or metacontrast masking. Longer length flanks, i.e. more energy, can yield weaker masking than equal length flanks for metacontrast masks (Figure 5). 2*25 contextual elements yield better performance than four lines in pattern masking (Figure 2).

**Spatial layout: Grouping**. On a stimulus description level, we propose that the complex spatial effects we reported hitherto can be best explained in terms of spatial grouping. In pattern masking, mask elements exert no influence on the target if they are grouped within an entity different from the target. For example, single contextual lines exert interference on the vernier, whereas this interference vanishes when the lines are grouped within an extended contextual grating (Figures 1 and 2; Herzog et al., 2004; Herzog & Koch, 2001; Herzog, Schmonsees, & Fahle, 2003a, b). On a neural description level, we showed that the proposed grouping effects can be mathematically modelled with simple differential equations mimicking the spatial processing in early cortical areas such as V1 (Hermens & Ernst, this volume; Herzog, Ernst, et al., 2003; see also Zhaoping, 2003). These models do not contain an explicit grouping operation and were not proposed to explain masking. Computer simulations with these models show that redundant elements, e.g. inner lines of gratings, are removed from further processing by dynamic lateral inhibition. In this respect, masking may be viewed as redundancy reduction (Reeves, this volume). It is important to note that ‘‘grouping’’ is a term of perceptual organization and may therefore be explained by several types of neural network models. Hence, it has to be seen whether existing mathematical models of masking (e.g. Bridgeman, 1971; Di Lollo et al., 2000; Öğmen, 1993) can capture the above effects when extended by appropriate spatial processing components (for the 2D model of Francis, 1997, no simulation results are available because of limited spatial resolution).

We propose that grouping also plays an important role in metacontrast masking. For short SOAs, the vernier offset can hardly be discriminated when it can be grouped within the flanking lines – as the single contextual elements lose their power when grouped within contextual gratings (Figure 2; see also Malania, Herzog, & Westheimer, 2007; Sharikadze, Fahle, & Herzog, 2005). When SOA increases, grouping breaks down by temporal cues and performance improves. To the best of our knowledge, there are only a few visual masking studies taking complex spatial aspects into account beyond low level variations such as varying mask size or the distance between target and mask contours. Williams and Weisstein (1984) showed that B-type masking occurs when the target appears as part of a 3-dimensional structure but A-type when not. More recently, Moore and Lleras (2005, Lleras & Moore, 2003) argued that masking depends strongly on whether or not the target and mask can be processed separately (see also Kahan & Mathis, 2002). 

It is surprising to see so few studies jointly investigating temporal and spatial vision even though the first goal of vision is the generation of a coherent spatial representation of the outer world that, as masking shows, is not created instantaneously. Spatial and temporal vision research belong together.
